# Changes in the Perception of Cemeteries as Public Spaces – Swiss Cemetery Visitors From 2002 to 2021

**DOI:** 10.1177/00302228241263133

**Published:** 2024-06-20

**Authors:** Harald Klingemann

**Affiliations:** 1 Bern Academy of the Arts, Institute of Design Research (IDR), 69477 Bern University of Applied Sciences, Bern, Switzerland

**Keywords:** cultural differences, diversity, burial, alternative landuse, user perspective, green space, long-term comparison

## Abstract

**Background:** A process of “emptying” can be observed in Swiss cemeteries. Urn burials are increasingly preferred to traditional interments, requiring much less space. Consequently, cemeteries are gradually transforming into park-like areas, triggering debates about proactive ways to use them as public spaces, rather than mourning sites. **Objectives and Method:** The study presented here probes for changes in the public perception of Bern cemeteries during the last two decades. The long-term analysis is based on a secondary analysis of interviews with 383 cemetery visitors in 2002 and a representative survey among 519 inhabitants of the city of Bern. **Results:** Tolerance for alternative land use increased further: Motivations for cemetery visits have shifted with a decline from 80% who mentioned ‘grave visit’ to 63% in 2021; ‘leisurely walk’ increased from 63% to 83%. High satisfaction with what cemeteries have to offer is mirrored in high values for perceived restorativeness in 2021.

## Analytical Cemetery Landscapes – the Conditions for a Long-Term Comparison

Societal change is reflected in our shifting attitudes to death and in the burial cultures associated with it (Walter, 2012). A town’s policy toward cemeteries is thus confronted with the challenge of responding adequately to modernising trends. Landscape planners and architects are increasingly engaging with environmentally friendly, citizen-friendly cemetery designs as a special instance of urban green spaces (Dlugozima & Kosiacka-Berk, 2020). But the multifunctionality of cemeteries (Säumel et al., 2023) harbours within it the potential for social conflict. According to Woodthorpe (2011), cemeteries may be characterised as ‘analytical landscapes’ on an emotional, commercial and community level. These levels are interlinked, as Woodthorpe explains by offering an example: “Concerned about the long term financial future of the site (cemetery) … The staff … have made moves to address how they could promote the cemetery as both a provider for bereavement services and a wider local community resource for education and recreation.” (Woodthorpe, 2011, p. 267). More recent studies have also considered the ‘demand’ side, in other words the user perspective, and have explored the acceptance or rejection of alternative uses for cemeteries (e.g. Al-Akl et al., 2018; Evensen et al., 2017; Goh & Ching, 2020; Grabalov, 2018).

The intensity of the conflict between the ‘cemetery as a place of mourning’ and the ‘cemetery as a resource for recreation and leisure’ depends on **assorted macro-level circumstances** that have rarely been addressed in the literature to date. The proportion of green spaces no longer in use for burial purposes is growing. This means that questions regarding possible alternative uses are becoming increasingly topical, to the point of open conflict occurring in cases where the gradual conversion of cemeteries into parks has been attempted (Klingemann, 2022). This development is closely linked to burial preferences, as we can see in the case study presented here of the three cemeteries in Bern in Switzerland, which are also typical of other Swiss cemeteries (Klingenberg, 2023).

**Figure 1.**
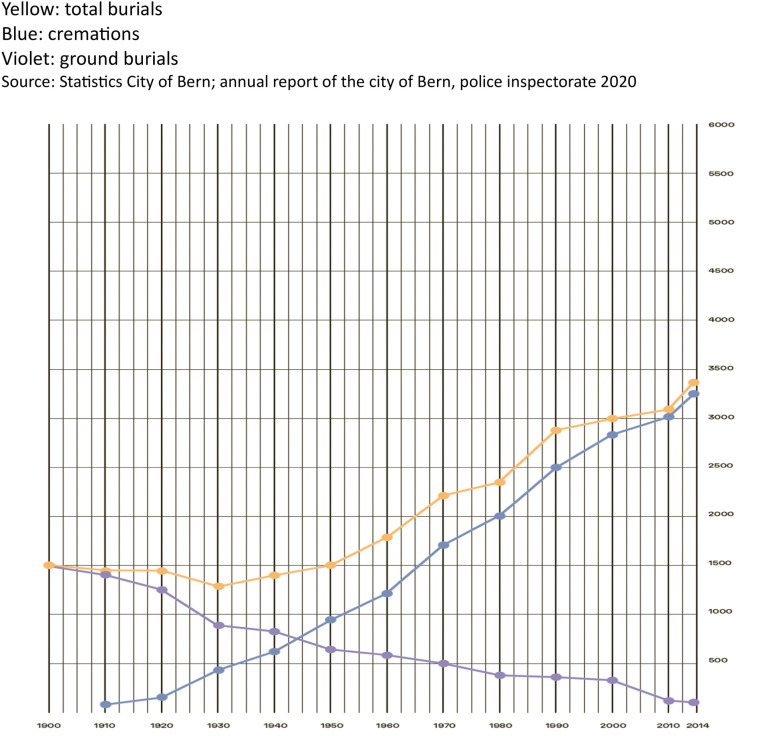
Burials city of Bern 1900–2014.

The most important factor in the utilisation of the land is the *cremation rate*. Urn burials only take up a fraction of the cemetery space needed for traditional burials. The higher the cremation rate, the more this leads to an ‘emptying’ of the cemeteries. The cremation rate drops in those countries where the proportion of the population that is Roman Catholic is higher (Maddrell, 2023), but is high in the case of Switzerland, standing at 85% as of 2019. Since the turn of the 21st century there has been a downward trend in the number of traditional burials. (Figure 1).

**Image 1.**
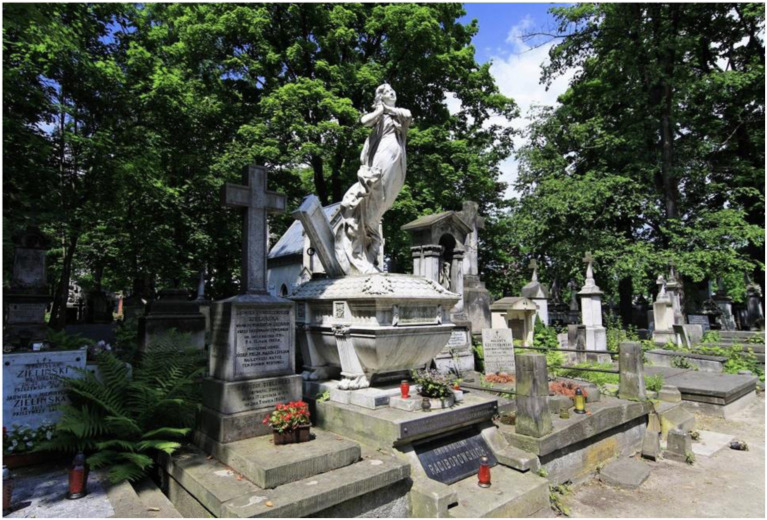
Powazski-cemetery Warsaw/Poland. Source: Warsaw tourist office. https://warsawtour.pl/de/powazki-friedhof/.

If we take a Catholic country such as Poland as an example, cremations accounted for just 24% of all deaths in 2016 (Cremation Society of Great Britain). This is in turn reflected in the amount of green spaces being freed up in cemeteries (Image 1).

**Image 2.**
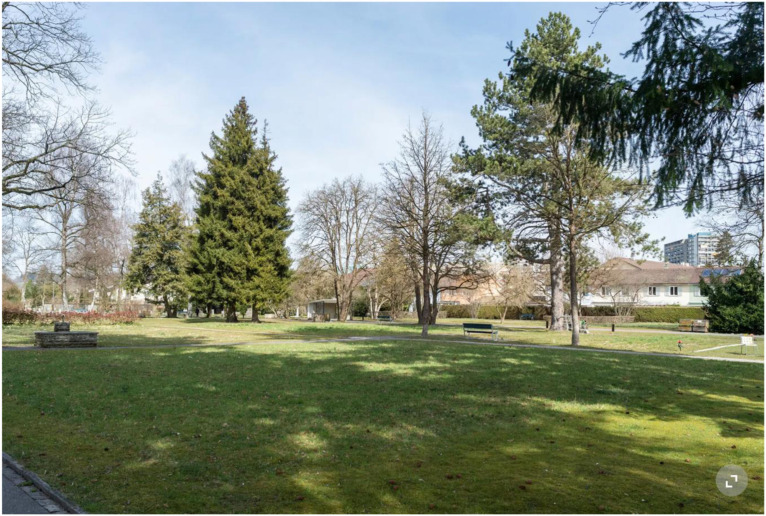
Bümpliz cemetery, Bern, Switzerland. Source: photo Franziska Rothenbühler; Berner Zeitung. https://www.bernerzeitung.ch/berner-gemeinden-werten-friedhoefe-auf-695574423314.

In countries like Switzerland where it is *not compulsory to bury the dead in cemeteries*, this ‘emptying’ process (Image 2) is further encouraged by the existence of cheaper options for *burial outside cemeteries*.

The parameters are thus clear for the case study presented below on the alternative use of the cemeteries of the city of Bern, and allow us to situate it in an international context. The following analysis focuses on the city of Bern’s perspective on the development of what visitors to its cemeteries imagined for the use of these spaces in 2002 and again in 2021, when also non-visitors were included. There have until now been no long-term comparisons, and these have been possible in the present case only thanks to fortunate circumstances (the detailed research report and the evaluation tools from 2002 are still available). The pre-history of the project is as follows.

In 2002, the project *Sepulkraldesign in der Modellregion Bern* (‘Sepulchral design in the model region of Bern’) was set up at the Bern University of Applied Sciences. Its aim was “to provide an empirical basis for the current debates on the use of these public spaces (cemeteries) and to outline in greater detail the expectations of cemetery visitors” (Kretz, 2003; project summary). The 2002 study raised a specific question regarding the extent to which people might be willing to use cemeteries as public spaces, and also addressed their putative wishes regarding burials, funeral services and the design of gravestones. The results of that study were set out in a detailed final report that provided a stimulus for further planning and attracted media attention (Kretz, 2003).

In their conclusion, the authors stated that this was the first time that Bern’s cemetery visitors had been asked for their opinion. Their study was undertaken in the spirit of a client-oriented cemetery policy that put both handed-down regulations and current offerings to the test. They identified a leitmotif running through the responses, namely the existence of tension between the collective on the one hand (whose public-orientated norms regarded cemeteries as a coherent, harmonious unit) and the wishes of the cemetery users along with the group interests of those involved in the funeral business on the other.

Over the ensuing two decades, the problem of cemeteries acquired further momentum. This was in part because of shifting burial preferences, an increase in religious and cultural diversity, but above all on account of a growing potential for conflict between different user groups. This conflict was already foreseeable back in 2002 and is even reflected in the title of the final report at that time: ‘Cemeteries for strolling or for mourning?’ The discussions about alternative uses for cemeteries reached a critical point in 2021 in view of the city’s medium-term plans to convert the Bümpliz Cemetery into a park. This conflict in the municipality and was analysed in greater detail that same year by means of a representative telephone survey of 519 residents of the city of Bern (Klingemann, 2022). This survey additionally addressed topics that had already been addressed in the survey of 2002. Besides exploring people’s tolerance towards the multifunctional redesign and use of urban green spaces, it was also possible for the first time – at least to a certain extent – to make comparisons between people’s opinions two decades apart: about their reasons for visiting the cemetery, about their needs and degree of satisfaction as users of the space in question, and about their attitude towards the alternatives available apart from burial in a cemetery. The present study can thus offer an overall picture of these shifting dynamics from the perspective of cemetery visitors, and against the background of innovations that have been implemented at the three Bernese cemeteries over the last two decades.

## Methods, Data

The **data collection in 2002** of the competence-building project ‘Sepulchral design in the model region of Bern’ was an *convenience sample* that *took place* during a single week from 3 to 10 September 2002. A total of 542 passers-by and visitors to the three cemeteries of Bern were approached for an interview, to which 363 agreed. The hours between 11 a.m. and 2 p.m. and again between 4 p.m. and 7 p.m. were chosen for the interview windows so that the sample might include both people in employment and those who were not working. It also meant that both visitors to the cemetery and passers-by who were there for other reasons might be taken into account. In the comparative analysis that is presented below, we refer to the results of the detailed final report of the 2002 study (along with the questionnaire used). We have been able to use as baseline the results as they have been reported in tabular form as frequencies or via correlations/analyses of interrelationship. Unfortunately, the original data set – namely the primary data from the interviews of 2002 – is no longer available.

The **follow-up study** was conducted in the summer of **2021** by gfs-Zurich/Market & Social Research as a representative telephone survey (CATI) of *n* = 519 adult residents of the city of Bern and Ostermundigen. Some of these telephone interviews, which lasted on average 18.7 minutes, took place via an online panel (*n* = 62). An important addition to this follow-up study was the *inclusion of potential users* who had **not** visited a cemetery in the previous two years. The 2002 questionnaire was used as a reference when designing the new one so that comparisons might be made. Individual questions nevertheless differ in some cases. This has to be taken into account when interpreting the comparative results.

Finally, it should be noted that the changes observed below that had taken place between the two survey dates of 2002 and 2021 can essentially be traced back to sociographic changes in the composition of the cemetery’s clientele (the age effect) that is independent of the shift in societal attitudes towards death (the cohort/generation effect) and also, ultimately, to the innovations that were implemented at the cemeteries **during** the period from 2002 to 2023 (the period effect). The **causes** of these changes can only be discussed within the context of an interplay between these different factors, also with the aid of additional sources of information and on the basis of practical experience. This is why the cemetery managers were asked in November 2021 to comment on the research report of the initial 2002 study (Kretz, 2003), in other words to bring to the table the **providers’ perspective on the dynamics of change**. Finally, the Head of Cemeteries & City Gardens provided an overview of the changes that had been made to the cemeteries between the initial survey and the follow-up study (September 2002 – July 2021) and between the follow-up survey and the present day (August 2021 – December 2023). These changes were taken into account when interpreting our results.

## Results

### Visitor Profile

If we compare the socio-demographic profile of cemetery visitors in 2002 and 2021, we can observe considerable shifts, even when we take the above-mentioned methodological reservations into account (see Table 1). The proportion of visitors who feel that they belong either to no religious community or to one that is ‘other’ has doubled (from 11% to 22%), while middle-aged people (40 – 64 years old) were more prominently represented in 2021 (57% as against 35%) and the over-65s only accounted for just under a quarter of visitors (cf. Table 1). At the same time, city transport links have clearly made cemeteries easier to reach. Half of the visitors went there on foot in 2021; in 2002, 38% of the interviewees stated that they lived near a cemetery. These trends are even more striking if we look at the **potential** visitors (i.e. non-visitors) in 2021. When compared with the actual cemetery visitors, the potential visitors are significantly younger (35% of the latter group were under 40 compared to 20% of the former) and either give ‘other’ as their denomination or belong to none (44% vs. 30%).

**Table 1.** Sociodemographic Profiles of Cemetery Visitors 2002 and 2021.

Characteristics	2002 Visitors N = 336	2021 Visitors N = 356	2021 Non-Visitors *N* = 163	Significance
(1) Religion				*p* < .008
Roman ccatholic	23%	20%	16%	
Protestant	63%	50%	40%	
Other	3%	%	19%	
No affiliation	11%	22%	25%	
(2) Sex				
Male	34%	46%	51%	
Female	66%	54%	49%	
(3) Age group				*p* < .005
<20 years old	4%	1%	1%	
20 to 39	23%	19%	34%	
40 to 65	35%	57%	45%	
>65	38%	23%	20%	
(4) Cemetery (interview/best known)				
Bremgarten	34%	37%	-	
Schosshalde	36%	41%	-	
Bümplitz	30%	22%	-	
(5) Cemetery proximity				
Neighborhood	38%		-	
Within walking distance	-	50%	-	

Legend: (3) 2002: estimated age group; 2021: exact age; (4) 2002: Cemetery where interview took place; 2021: crossed or visited at least one Bern cemetery during the last two years. (5) 2001: «Do you live close to a cemetery?’ 2021: «how do you reach the cemetery usually (on foot, other means of transportation).

### Visitor Requirements and Expectations

#### Visitor Motivation, Reasons for Visiting

Over the period in question, people’s motivation for visiting a cemetery underwent several significant shifts. In 2002, 80% of respondents still mentioned “visiting a grave often or occasionally”; this proportion fell to 63% in 2021. “Going for a walk” as a reason for visiting a cemetery was the frontrunner in 2002, given by 83% of people, compared to 63% in 2021 (see Table 2). The importance of more pragmatic reasons is also worthy of note. For example, in 2002, three quarters of those asked said they would never use a cemetery simply as a transit route, but in 2021 only two-thirds of visitors said that they were still reluctant to do so. Changes in transport planning could also have played a role in this, as might issues such as the private use of the flower shop in the cemetery (only surveyed in 2021; 31% of participants use it) or participating in guided tours or events (also only surveyed in 2021; 20% of people responded in the affirmative).

**Table 2.** “How Often Have You Visited the Cemetery Because of …”.

Reason	Cemetery Visitors 2002 (*n* = 357)		Cemetery Visitors 2021 (*n* = 313)	
Frequency	Never	Rarely to Often^a^	Never	Rarely to Often^a^
Quietness, contemplation	28%	72% (2)	29%	71% (3)
Nature reflection	31%	69% (3)	24%	76% (2)
Stroll	37%	63% (4)	17%	83% (1)
Using as a passage	73%	27% (5)	35%	65% (4)
Meeting point	79%	21%	74%	26%
Visiting a grave	20%	80% (1)	37%	63% (5)
Work break	85%	15%	83%	17%
Flower shop for private use	Not asked	Not asked	69%	31%
Event, guided tour	Not asked	Not asked	80%	20%

^a^The research report 2003 (no primary data) indicates only a summarized category ‘rarely to often’. As to the 2021 survey (primary data available), ‘leisurely walk’ also ranks in top position with 19% of respondents indicating ‘often’.

#### Attitudes to Specific Alternative Uses

As the title of the 2002 report on the results of the initial survey intimated (‘Cemeteries for strolling or for mourning?’), planners were already interested in the extent to which people would accept alternative uses of cemeteries as urban green spaces, and what scope might exist for extending such alternatives. Visitors were accordingly asked to give their opinion on a list of assorted activities and services at the cemetery – such as how appropriate or inappropriate they found them. These questions were largely replicated in the follow-up survey of 2021 and were also expanded to include several further aspects. Our comparative analysis therefore focuses on whether the *extent* of people’s acceptance of alternative uses has changed from 2002 to 2021 and, linked with this, whether there have been any shifts in how such acceptance might be *linked* to motive-specific visitor frequency.

In our comparative interpretation of the results, we have to take into account any changes that have *already been realised* by the cemetery management during the period in question. The Head of Cemeteries & City Gardens provided us with the appropriate information and commented on the list of alternative uses from the perspective of “what had been significantly altered or introduced at the three cemeteries between September 2002 and the time of the survey in July/August 2021”. These comments will be incorporated as background information in our below comparison.

A glance at Table 3 shows a consistent, **significantly higher acceptance of alternative uses between 2002 and 2021** (Table 3, columns 4 and 6).

**Table 3.** Approval of Specific Leisure Activities and Options on Bern Cemeteries 2002 and 2021.

	Phone Interviews (CATI)			Personal Interviews on Site	
	2021	2021	2021	2002	
	Total Sample	Non-Visitors Last 2 Years	Visitors Last 2 Years	Visitors	
	(N = 519)	(N = 142)	(N = 377)	(N = 383)	
(Rather or Very ….)	Appropriate	Appropriate	Appropriate	Wording 2002/2021	Appropriate
Quiet games (e.g. chess)	67%	67%	67% +27%	Same	40%
Grazing sheep	67%	60%	60%+11%	Same	49%
Cultural events	54%	46%	57% +30%	Same	27%
Reclining benches	51%	51%	61%	2002 not asked	—
Yoga	51%	45%	53%	2002 not asked	—
Exchange of thoughts with a small beer	42%	38%	43%	2002 not asked	—
Jogging (non specified)	38%	38%	38%	Jogging paths (specified)	11%
Picnic (non specified)	39%	40%	39%	Picnic places (specified)	14%
Lively games (e.g. Boule)	27%	28%	26% +13%	Same	13%
Cycling (non specified)	24%	25%	23%	Cycling paths (specified)	11%

The dynamics of change may be described individually as follows:

Whereas 27% of visitors in 2002 found **cultural events** at the cemetery *“fairly appropriate or very appropriate”*, this figure rose to 67% in 2021! Such a massive increase indicates considerable demand potential; according to the cemetery management, thus far only special events have occasionally been held at any of the cemeteries during the period in question (theatrical performances, readings, participation in ‘museum nights’, open days).

In second place in the dynamics of change is an increase of 27% in the acceptance of ‘**quiet games**’; ‘**lively games**’ enjoyed an increased in acceptance among 13% of the survey participants. As far as the latter games are concerned, pétanque has been allowed at the Mergelplatz at the Friedbühl^1^ area of Bremgarten Cemetery since 2017; no chessboards or similar are available there, but would be welcomed by 67% of visitors.

‘**Grazing sheep**’ enjoyed the next-biggest increase in acceptance, with 11%. Allowing sheep to graze was already accepted by a high number of people back in 2002, namely 49%; this rose to 60% in 2021. There was a significant consensus here, both on account of the Christian symbolism of the animal, and because of their function as ‘ecological lawnmowers’. In fact, sheep were introduced to all the cemeteries on a rotating basis in 2015, but since 2018 they have been present only at the Bremgarten Cemetery. The introduction of sheep was apparently experienced as something positive, and a majority of visitors would presumably welcome an expansion of their use. As for the rates of change for a group of further alternative uses, we have to take into account the fact that there were *divergences in the questions asked*.

In 2002, 14% of people felt that it was fine to set up picnic *areas*, but 39% were in favour of ‘**picnicking**’ as an activity in 2021. By the time of the second survey, however, the increase in interest in picnics had already been partially satisfied. From roughly 2000 onwards – thus at the time of the initial survey – movable benches, chairs and other such furniture were already available at all the cemeteries. There is no ban on picnicking, and waste can be disposed of in the existing bins on site.

In the 2002 survey, people were also asked if they would be in favour of creating cycle *paths*. Eleven percent said ‘yes’, and by 2021, more than twice as many (23%) said they’d generally approve. In fact, **cycling** remains prohibited in cemeteries, though offenders are not penalised across the board. The attitudes to jogging *paths* were similar in 2002, with 11% of visitors finding them appropriate. But **jogging** in general was already regarded as perfectly acceptable by 38% of visitors in 2021. By comparison, observational studies at Scandinavian cemeteries (in Malmö) have come to the conclusion that “Jogging is an essential part of the activities taking place in each of the cemeteries and does not have any visible conflict with the other functions of the cemetery” (Grabalov, 2018, p. 78). When it comes to jogging, picnicking and cycling as alternative uses for cemeteries, we may plausibly assume that the actual figures for those in favour were lower than stated here because the associated follow-up questions in 2021 set a lower threshold. Nevertheless, especially with regard to the 39% acceptance of picnicking, these changes indicate *increased* tolerance.

The report on the results of 2002 states with regard to changes of use: “The level of approval is high for those proposed changes in use that are quiet and passive in nature, but still remain below 50%” (Kretz, 2003, p. 16). The follow-up survey of 2021 puts this finding into perspective, demonstrating significantly *higher* approval rates of over 50%. **This trend towards ‘quiet, reflective recreation’** – which can also be observed at cemeteries in Norway and Sweden (Nordh et al., 2023) – is further validated if we also consider the degree of acceptance of ‘*yoga*’ and ‘*reclining benches*’ (questions that were only asked in 2021), whose approval rates are 53% and 61% respectively. In the period under comparison, yoga was not prohibited and the cemeteries were also used for this purpose. Visitors to the Bremgarten Cemetery who are interested are referred to the offerings at the Friedbühl area at the outskirts of the cemetery. However, no designated recliner benches have been installed, so there is room for improvement here. It remains open to what extent the option of **‘exchanging ideas over a small beer’** belongs in this category of alternative uses (it is accepted by almost half of the visitors, namely 43%). Current regulations do not provide for a ban on alcohol. And with the sole exception of several adolescents at the Bümpliz Cemetery, no problems in this regard have been observed.

Finally, if the group of **non-visitors** to the cemetery is taken into account, the acceptance rate is by no means higher as had been assumed in the final report of the 2002 survey (Kretz, 2003, see p. 58 below). In some cases, the tolerance level was actually somewhat *lower* (see Table 3, cols. 3 and 4). At the same time, analyses of attitudes towards converting the Bümpliz Cemetery in Bern into a park show that almost 50% of non-visitors *generally* tend to a significantly higher rate of approval than cemetery visitors (Klingemann, 2022, p. 5). However, this willingness to change is clearly not accompanied by a more open assessment of **specific** conversion options, and there is little coherent association here with more traditional images of a cemetery.

A further step in the analysis involved once more examining the extent to which an acceptance of specific alternative uses is related to the respective frequency of visits to a cemetery. Corresponding correlation analyses of the 2002 survey came to the conclusion “that visitors to gravesites perceive every other use as less appropriate than do other visitors to the cemetery. With regard to tolerance for ‘grazing sheep’ and ‘cultural events’, there were somewhat smaller differences of opinion” (Kretz, 2003, p. 24).

The results of the 2021 survey reveal a different picture in two respects. *First*, alternative uses continue to be rejected more strongly by those who visit graves more regularly, but to a much *weaker* degree (‘picnicking’, ‘jogging’, ‘boules’, with the exception of ‘quiet social games’), or to a degree that is *no longer significant* (‘sheep’, ‘culture’, ‘cycling’), as is shown by our comparison of the correlation coefficients (see Table 4, with comparative figures in column 2). The idea that ‘exchanging ideas over a small beer’ is acceptable at a cemetery – which was only asked in 2021 – does not covary with the frequency of people’s visits (43% find this fairly acceptable or very acceptable), while ‘benches’ and ‘yoga’ receive a mild rejection.

**Table 4.** Declining Opposition of Grave Visitors Towards Alternative Use Covariations (Pearson Corr.) 2002 and 2021.

Degree of Approval of Alternative Use	Visiting a Grave - Frequency
	Pearson Corr 2002/2021
Culture events	−.15**/−.11*
Picnic	−.27**/−.21**
Jogging	−.21**/−.17**
Cycling	−.27**/−.11*
Lively games	−.19**/−.17**
Yoga	Only 2021/−.16**
Reclining benches	Only 2021/−.16**

Legend: ** sig.< .001; * sig. < .05.

*Secondly*, visitors to gravesites were the focus of the 2002 analysis, but 2021 saw major changes in the relative importance of their reasons for visiting the cemetery. In the frequency ranking for people’s motives for coming to the cemetery, ‘visiting a grave’ drops from 80% to 63%; the frontrunner in 2021 is ‘taking a walk’, with 83% (2002: 63%), followed by ‘contemplating nature’ with 76% (2002: 69%) (see Table 2 above). These motives do *not* imply any conflict with the aforementioned options for change, with the exception of a mild rejection of lively and quiet social games (rp = −.12; rp = −.11) (cf. Table 4 cols 3 and 8). These shifts in the motivation profile of visitors indicate a reduction in any resistance to reutilisation plans at the cemeteries.

### General Customer Satisfaction With Cemetery Services and Perceived Restorativeness

In the 2002 survey, 90% of respondents indicated a generally high degree of satisfaction with the various services offered at the cemetery, including planting at gravesites and burial ceremonies (Kretz, 2003, p. 25). Only 12% were dissatisfied with the range of plants offered, and found that the signage system for finding other, older, ‘famous’ gravesites was in need of improvement (Kretz, 2003, pp. 25, 26).

While this aspect was not assigned the same weight in the 2021 survey, the latter asked the open question “What was lacking on your last cemetery visit?”, which we might use for an approximate comparison. 88% of visitors stated that nothing had been lacking, which overall reflects a relatively high level of satisfaction with the cemeteries, as had been the case in 2002. The remaining 12% of visitors in 2021 wanted improvements similar to those requested in 2002. At the Schosshalden Cemetery, people primarily mentioned problems with the infrastructure (benches, parking spaces, opening hours, a ban on dogs), while assessments of the other two cemeteries focused on aspects such as planting, signage and burial issues (e.g. new themed graves).

What has contributed to the overall satisfaction of cemetery visitors from 2002 to 2021, and what has helped to increase their degree of satisfaction?

In order to help answer this question, we also analysed for the first-ever time the increasing importance of reasons other than visiting gravesites for those who come to a cemetery, such as walking or being close to nature; we also examined the *impact* of these activities on the state of mind of the visitors. We were able to draw specifically on research that perceives the general impact of the landscape on people as something of restorative value (e.g. Bauer et al., 2021; Dzhambov et al., 2019) and on studies that apply such an approach to cemeteries (Dlugozima & Kosiacka-Berk, 2020; Lai et al., 2020). According to Attention Recovery Theory (Kaplan, 1995), the positive impact of natural environments can help us in a process of regeneration, enabling us to focus our attention better, reduce stress (Huang et al., 2020) and improve our well-being (Home et al., 2012) if these environments have the following characteristics: “objects or events demand attention without the observer having to exert any effort (‘fascination’); the environment allows one to erect a mental distance between oneself and the tasks and goals that are usually pursued (‘being away’); the environment is perceived by the observer as coherent in itself and substantial in its ‘scope’; and the environment is consistent with one’s own ideas and goals (‘compatibility’); the environment is perceived as ordered and ‘coherent’ (Bauer et al., 2021, p. 22). These criteria have been used to create the *Perceived Restorativeness Scale* (Hartig et al., 1997; Pasini et al., 2014) that is utilised in research for the comparative assessment of the restorative potential of different landscapes, parks, gardens and also cemeteries (Lai et L. 2020). The German-language version of this Scale by Schönbauer (2013) was used for the follow-up survey of 2021; it has 12 questions (see Table 5, col. 1). The summated scale ‘perceived restorativeness value 12’ (PR12) created using these questions is used in the below analysis and has good statistical properties with a Cronbach alpha value of .853.^2^

**Table 5.** Subscales and Items of the Perceived Short Restorativeness Scale.

«How Much do you Agree With the Following Statements When you Think of Your Impressions During Your Last Visit to the Cemetery (During the Last Three Months? »	Mean Scale 1 – 5
Fascination	
«There is much to explore and discover here»	3.43
«My attention is drawn to many interesting things»	3.22
«This place has fascinating qualities»	3.57
«I want to get to know this place better»	2.89
Coherence	
«There is a clear order in the physical arrangement of placs lke this»	4.00
«In places like this everything seems to have ist proper place»	4.19
«It is easy to see how things are organized here»	4.00
Compatability	
«I can do things I like here»	3.53
«Being here suits my personality»	3.30
Being away	
«Coming here helps me to get relief from unwanted demands on my attention»	3.50
«Spending time here gives me a break from my day-to-day routine»	3.34
Scope	
«This place is a world of ist own»	3.79

Table 5 (cols 2–4) indicates *an overall high restorativeness value for Bern’s cemeteries*, especially with regard to ‘coherence’ (mean values 4 and above). Sub-aspects of ‘fascination’, on the other hand, are somewhat weaker (‘curiosity’ and ‘interesting things’ having x = 2.89 and x = 3.22 respectively).

The next step was to examine the extent to which the frequency of the visit for the reason in question was associated with the perceived degree of restorativeness.

Table 6 shows that the perceived restorativeness increased significantly with the number of visits for all reasons – with one exception, namely among those who came to the cemetery to visit a specific grave.

**Table 6.** Covariance (Pearson Corr) of Perceived Restorativeness (WE12) and Frequency of Reason to Visit Among Cemetery Visitors (Durings Last Three Months) 2021 (*n* = 227).

Reason - Frequency-	Pearson Corr WE12 and Frequency of Reason to Visit	Significance (2-Tailed)
Quietness, contemplation	.436	<.001
Nature reflection	.448	<.001
Stroll	.366	<.001
Using as passage	.159	<.017
Meeting point	.305	<.001
Visiting a grave	.120	n.s
Work break	.137	<.039

At first glance, this finding is consistent with the results of the study by Lai et al. (2020), who compared the perceived restorativeness value of parks and cemeteries in Scotland and similarly found that environmental characteristics had no impact on perceived restorativeness among cemetery visitors who had a loved one buried there. By contrast, the follow-up study of 2021 presented here shows *no* differences in perceived restorativeness between people with a loved one buried in the cemetery (*n* = 117) and those without (*n* = 94); they have almost identical mean values (x = 3.56, sd = .757 and 3.55, sd = .659 respectively).

These apparent contradictions are resolved, however, if the *frequency* of visits is understood as a multiplier for exposure to the cemetery environment and categorised accordingly, depending on the reason for the visit. Restorative effects can only be amplified when the person concerned is *moving about* in the natural environment. In their study on gardens, Bauer et al. (2021) found “that moving about is particularly important for high stress levels, while both moving about and spending time in green spaces are important for reducing moderate stress” (Bauer et al., 2021, p. 29).

A glance at Table 6 shows that the more people visit the cemetery for purposes of movement (strolling etc.), the more they become aware of its restorativeness. A holistic perception of the cemetery environment is thus barely favoured by short or occasional, ‘stationary’ visits. Work breaks, using the cemetery as a transit route and visits to graves can be categorised as belonging to this group.

From a planning perspective, there is a practical aspect to asking what characteristics of the natural environment have an impact on the perceived restorativeness. Research findings have indicated that biodiversity (e.g. the number of plant species present) (Kowarik et al., 2016; Massoni et al., 2018; Young et al., 2020), good paths, the ‘pleasantness’ of the landscape, aesthetics and safety (Lai et al., 2020) all have a positive influence on perceived restorativeness.

**We may assume that changes were made to Bern’s cemeteries during the period under observation (2002 to 2021) that will have help to achieve the high level of perceived restorativeness by the time of the follow-up survey in 2021**. In terms of *biodiversity* (Löki et al., 2019), themed graves for urns were introduced between 2013 and 2018 whose topics were ‘roses’, scented plants, shrubs, trees, flowers, blossoms and butterflies; these were complemented by the introduction of the new planting schemes ‘autumn magic, summer and autumn specials’, as well as specific areas designated to promote biodiversity – such as stone walls suitable as a habitat for small lizards. The cemeteries were also made more attractive for observing rare birds – which is one way of making a cemetery more interesting as a place where there are things to discover (-> the dimension ‘Fascination’ on the restorativeness scale). Ultimately, we may assume that there was a link between the introduction of new, uniform signage (steles, maps, signposts, labelling) for the three cemeteries (2017–2020) and the ‘coherence’ dimension of the restorativeness scale.

### Cemetery Burials and Alternatives

The research report on the visitor survey of 2002 already reached the conclusion that “The results reveal a considerable interest in new forms of burial, also outside the cemetery” (Kretz, 2003, p. 44). Participants were given a selection of possible answers (with multiple answers possible) to the question “When you think of your own burial, how appropriate would you find the following types of burial?”. A ‘tree burial’ was considered possible by 50%, while 48% were able to imagine being buried ‘outside a cemetery (ashes dispersed in Nature)’ and 21% ‘ashes dispersed in my own garden’. The other options also included ‘a dual burial with my partner’, which 57% regarded as ‘appropriate in principle’. The high degree of acceptance of these types of burial already revealed potential for the future, even if these alternatives were not yet particularly relevant in practice back in 2002. In 2021, the corresponding question was framed with greater clarity, and the choice was specified between a cemetery – without mentioning any particular form of burial – and ‘not in a cemetery’: “When you think about your own burial, would you like to be buried in a cemetery?” (open answer). This **type of question** shows that while 2002 saw an as-yet vague acquiescence in the idea of a burial outside a cemetery, by 2021 a total of 24% (*n* = 89) cemetery visitors preferred the concrete alternative of such a burial for themselves. Among non-visitors to the cemetery, that figure is actually 41% (*n* = 58), which is a significant group difference (*p* < .001). What is striking in this context is the relatively high proportion of respondents who were unable to decide on an answer to this question, namely 17% (*n* = 64) of the cemetery visitors and 23% (*n* = 32) of the non-visitors. This could suggest a resistance to dealing with the idea of one’s own death and/or a degree of ambivalence, given the broad selection of funeral options.

Survey participants who prioritised a burial outside a cemetery were then posed an open question about what specific ideas they might have in this regard. Almost three-quarters of those unwilling to be buried in a cemetery expressed a wish for their ashes to be scattered in nature. One subgroup of ‘in nature’ left this at simply ‘somewhere outside’, ‘ashes scattered in nature’; a second subgroup (‘ashes scattered at a specific natural location’) indicated the kind of natural landscape they desired for the scattering (in the air, the mountains, a forest, a field, a river, the sea).

Finally, the ‘social connotation’ category included references to a *place with personal significance* (e.g. “in my homeland, my garden, at home under a tree, in the sea near Ibiza, where I was born”), a desire to do something for the public good (such as a “donation to the University of Bern for medical training”) and delegating decisions on the type of burial to one’s family (“kids will decide”; “I leave it up to my daughter”; “relatives should decide”).

The numbers in these cases are low, with hardly any difference between visitors and non-visitors to the cemetery, though the former emphasise the ‘social connotation’ slightly more, and tend to have more specific notions about what they want (see Table 7).

**Table 7.** Out of Cemetery Burials and Preferences for Style of Nature Burials.

	Non-Visitors 2021 (*n* = 142)	Visitors 2021 (*n* = 377)
My own burial not on a cemetery	41% (58)*	24% (89)*
If not on a cemetery, additional response to preferred style of nature burial		
Non-specified nature burial	25% (14)	20% (18)
“Somewhere outside”		
“In nature”		
“Scattered in the air”		
Burial with a social connotation	19% (11)	26% (23)
“In my homeland”		
“Kids will decide”		
“Hospital donation”		
Nature burial - location preference	29% (16)	34% (30)
“Water, river, sea”		
“Woods, tree”		
“Mountain, field, hills”		
Open, not decided yet	30% (17)	20% (18)
	100% (58)	100% (89)

Comment: *Sig <.001.

Of those survey participants who are cemetery visitors and would like to be buried in one, 54% would prefer a cemetery in the city of Bern; in this category among the non-visitors, almost a quarter are undecided and only 28% of them would specifically like to be buried in a cemetery in the city. There is no significant difference between the participants’ specific cemetery preferences, though the Schosshalde Cemetery pips the others to the top of the list with 40% among non-visitors and those who want to be buried in a cemetery.

## Discussion

So are cemeteries for strolling or for mourning? That’s a false alternative! They’re for strolling and *also* for mourning. This is how we ought to answer the question posed by the original study, in view of the results presented here of our comparative study over the period of 19 years. The development dynamics of the past 20 years have been characterised by an increasing emphasis on the multifunctionality of cemeteries as green, urban spaces and by a greater focus on the needs of cemetery visitors.

However, we should note at the outset that certain **limitations** must be taken into account when interpreting the survey results. Our comparative analysis is based on published results and secondary data from the 2003 research report and thus did not allow for a statistical approach. We are also dealing with a representative sample in the 2021 survey, whereas the 2002 survey was based on an convenience sample, albeit using selected time windows. What’s more, the brief telephone survey in 2021 was unable to cover further aspects such as ‘funerals’ and ‘gravesites’, nor was it able to include economic considerations that had been thematised extensively in 2002. This means that we cannot make any statements about trends here, but must instead point to the possibilities offered by future research. Finally, we can only guess about the actual impact of the COVID pandemic on the results and the dynamics of change. The parks of the city of Bern remained closed from March 2020 to May 2020, but access to cemeteries remained unlimited, the only restrictions being on the number of people allowed to attend burials. We may therefore assume that cemeteries were more often used as green leisure spaces, and that this trend towards using them for recreational purposes has intensified, with the city meanwhile becoming even more densely populated.

While individual results may be up for discussion given this background, we may nevertheless assume the existence of a valid dynamic of change because practically every individual aspect points in the same direction. They reflect an overall trend towards greater tolerance and openness, in line with the motto ‘Cemeteries belong to everyone’ (73% agreement in the overall sample of 2021).

### The Individual Results

The socio-demographic profile of cemetery visitors had already changed between 2002 and 2021. There was an increasing presence of middle-aged groups (40–65) and of people who did not feel that they belonged to any specific religion or denomination (11% in 2002 as against 22% in 2021).

The increasing relevance of age and of generational and denominational affiliation is further illustrated by the differences between visitors and non-visitors in 2021. The latter group was significantly younger than the former (34% of non-visitors as against 19% for visitors belonged to the 20–29 age group) and did not belong to any of the large religious communities (19% vs. 8%). It thus seems that the cemeteries had been in a position to address this particular ‘demand potential’ to at least some extent.

We can also observe a greater sense of **‘balancing out’ when it comes to people’s motives for visiting** a cemetery. Whereas ‘frequent’ and ‘occasional’ grave visits lost their dominance (down from 80% to 63%), ‘taking a walk’ and ‘observing Nature’ saw an increase (from 63% to 81% and from 69% to 76% respectively).

In 2002, we saw a consistent dismissal of any changes in use, the more frequently people came to visit gravesites. But this reduced greatly by 2021 with regard to the respective correlation coefficients (such as lively games and picnicking), or at least gave way to a more tolerant attitude (no significant correlation in 2021) towards, say, cultural events and cycling.

When we compare 2002 and 2021, we see that a reduction in resistance to alternative uses for cemeteries has gone hand in hand with a significantly higher level of acceptance. If we focus on such increases in these dynamics of change, then we can observe the strongest rise in the approval of cultural events (from 27% to 57%), followed by ‘quiet social games’ (from 40% to 67%) and ‘grazing sheep’ (from 49% to 60%). The focus on quiet, passive changes in cemetery use in 2002 was more pronounced in 2021, when new options were also taken into account (yoga, reclining benches). At the same time, however, these trends also correspond to an increasing tolerance (albeit at a lower level) of alternative uses for cemeteries that involve greater physical and social activities, even when we take into account the slight divergences in the questions of 2002 and 2021. We can once more observe how user requirements balance out. This offers the cemetery administration *greater room for manoeuvre* to be more proactive in meeting the shifting needs of the public, such as promoting cultural events and quiet social games, installing recliner benches and reintroducing grazing sheep at all the cemeteries. The limited resources at their disposal mean that there are clear limits to such plans, which in turn makes it important to set priorities. However, resistance from cemetery users can no longer be used to justify the status quo.

### Increasing Diversity and a Shift in Values

The trend towards *alternative burials outside cemeteries*, which was already a matter of firm conjecture back in 2002, would seem to have grown. When given a choice, 24% of cemetery visitors in 2021 (and 41% of non-visitors) would prefer a nature burial outside any cemetery. Back in 2002, multiple responses were offered to a less specific question about the degree of ‘appropriateness’ of different forms of burial (48% found a burial outside a cemetery appropriate, along with other possible answers). When we look at the results of 2021, their variance and diversity are striking. There is a degree of uncertainty – 17% of visitors were unable to make a decision, or had not yet made one – while those in favour of a nature burial can be divided into subgroups with specific ideas about the extent to which nature overall should play a role, the location for scattering one’s ashes, and social connections.

Finally, from the perspective of the city’s authorities for cemetery and green-space management, the question arises as to just how they have succeeded over time in adjusting their offerings to meet the shifting needs of cemetery visitors as reported here. In 2002, an overall high level of satisfaction with the cemeteries’ assorted services was registered, with only 12% calling for improvements – such as in matters of signage and planting (Kretz, 2003, p. 25). The 2021 follow-up survey shows the same percentage of responses to the related question “What was lacking on your last cemetery visit?”. The ongoing high level of overall satisfaction among 88% of visitors indicates that the cemetery management had largely recognised the shift in visitor requirements with a greater emphasis on experiencing nature and towards a greater, secular use of the cemetery for leisure purposes, and that they had borne this in mind (at least implicitly, if not systematically) when reorganising the cemeteries. These findings were given empirical support in 2021 when the **perceived restorative value of the cemeteries was registered**: The scale that is generally used to record the impact of landscapes in different contexts showed a uniformly high level of perceived restorativeness for all three Bernese cemeteries. When specific gravesite visits are excluded, the perceived restorativeness of cemetery visits increases significantly in line with the rising frequency of visits for all other reasons. This is associated with the different levels of ‘immersion in the cemetery landscape’/exposure and ‘movement’ around the site.

It seems plausible that the restorativeness potential has been enhanced significantly over the past two decades through assorted changes to the cemeteries. We can assign these changes, after the fact, to the five dimensions/aspects of perceived restorativeness:

◦ A new, uniform signage system introduced in 2017–2020 -> perceived restorativeness -> ‘**coherence**’ (*It is easy to see how things are organized*).
◦ Promotion of biodiversity -> perceived restorativeness -> ‘**scope**’ (*a world of its own*)
◦ More cultural events, birdwatching - > perceived restorativeness -> ‘**being away**’ (*a break from my day-to-day routine*).
◦ New planting programmes; themed graves for urns, 2013 – 2018 - > perceived restorativeness - > **fascination** (*much to explore and discover*)
◦ Bremgarten cemetery 2017; flower shop/plans for park and restaurant zoning of an activity area at the periphery of the cemetery ('Friedhbühlanlage')-> perceived restorativeness -> ‘**compatibility**’ (*I can do things I like here*)

With regard to this last point, Nordh et al. (2023) assume, based on their observations in Scandinavian cemeteries, that “earmarking activity zones can help avoid conflicts between the different users”.

It would seem a good idea to consider using the concept of perceived restorativeness as a systematic framework for making future changes and to use it wherever possible to monitor them, thereby making comparisons possible with other urban green spaces. In this context, one should examine whether it might be feasible, for a reasonable outlay, to conduct surveys of the needs of cemetery visitors (and non-visitors) more systematically and more regularly in future, not just every 19 years. Local online panels might be an option here.^3^ It might also be conceivable to set up limited *experiments* in a ‘pilot cemetery’ and evaluate them afterwards: such as installing a large chessboard in a cemetery, and observing how this particular innovation is received and utilised. Ultimately, it is conceivable that a ‘cemetery observatory’ might be established, to be staffed by experts from various fields, possibly also from outside the Canton of Bern, who would be able to recognise new developments in good time. Their task would involve considering cemetery strategies for the future that take into account people’s different perceptions of what a ‘public space’ can be (Grabalov & Nordh, 2022). A citizen-friendly cemetery policy with a view to people’s shifting needs would mean staying in contact with clients located both close to the cemetery and far away from it, if we are to adjust the cemetery services offered in a manner that is both systematic and evidence-based.

## Notes

1. In 2017, out of consideration for visitors to the Bremgarten Cemetery, a transitional zone was created between the active burial grounds and the new neighbourhood square on the former site of a market garden. This transitional zone is located in the abovementioned southern part of the cemetery grounds.

2. This value expresses the degree of agreement between the individual questions and assesses whether they actually measure the relevant construct, in this case ‘perceived restorativeness’.
